# Data Valuation Algorithm for Inertial Measurement Unit-Based Human Activity Recognition

**DOI:** 10.3390/s23010184

**Published:** 2022-12-24

**Authors:** Yeon-Wook Kim, Sangmin Lee

**Affiliations:** 1 Department of Electrical and Computer Engineering, Inha University, Incheon 22212, Republic of Korea; 2 Department of Smart Engineering Program in Biomedical Science & Engineering, Inha University, Incheon 22212, Republic of Korea

**Keywords:** data valuation algorithm, meta-reinforcement learning, deep learning, transformer, convolutional neural network, human activity recognition, inertial measurement unit

## Abstract

This paper proposes a data valuation algorithm for inertial measurement unit-based human activity recognition (IMU-based HAR) data based on meta reinforcement learning. Unlike previous studies that received feature-level input, the algorithm in this study added a feature extraction structure to the data valuation algorithm, and it can receive raw-level inputs and achieve excellent performance. As IMU-based HAR data are multivariate time-series data, the proposed algorithm incorporates an architecture capable of extracting both local and global features by inserting a transformer encoder after the one-dimensional convolutional neural network (1D-CNN) backbone in the data value estimator. In addition, the 1D-CNN-based stacking ensemble structure, which exhibits excellent efficiency and performance on IMU-based HAR data, is used as a predictor to supervise model training. The Berg balance scale (BBS) IMU-based HAR dataset and the public datasets, UCI-HAR, WISDM, and PAMAP2, are used for performance evaluation in this study. The valuation performance of the proposed algorithm is observed to be excellent on IMU-based HAR data. The rate of discovering corrupted data is higher than 96% on all datasets. In addition, classification performance is confirmed to be improved by the suppression of discovery of low-value data.

## 1. Introduction

Deep learning algorithms have been used in various fields [1,2,3]. In the field of computer vision, deep learning algorithms are used to perform multi-modal learning to obtain useful information from images and texts, images and speech, and images and sensor signals [1]. In the field of audio analysis, deep learning algorithms are used for automatic speech recognition, audio enhancement, and audio generation [2]. In the field of natural language processing, deep learning algorithms are used to perform sentiment analysis and machine translation [3]. The training of these deep learning algorithms tasks requires large amounts of data. Using sufficient data during training prevents overfitting and enhances generalizability. Therefore, deep learning algorithms are widely used for big data analysis [4,5,6]. Unlike machine learning models that require handcrafted feature engineering, deep learning models can extract features and rules from data and output the desired signals or labels [7,8,9]. Therefore, both model structure and quality of training data play important roles in improving the performance of deep learning-based models.

The quality of training data is often degraded for various reasons. In the case of sensor data, artifacts of the sensor device or noise caused by the environment can degrade signal quality [10,11]. Moreover, manual labeling of collected data may be erroneous owing to mistakes or insufficient information [12,13]. Data collected via crawling may be unintentionally collected or incorrectly labeled [14,15]. Data labeled via crowdsourcing can also include labeling errors owing to human subjectivity or mistakes [16]. In turn, low-quality data of the aforementioned types degrade the model performance [17]. The challenge lies in the fact that obtaining high-quality data is time-consuming and expensive [14].

Several studies have implemented robust models, even with low-quality data, to overcome this limitation [18,19,20]. Moreover, studies have been conducted to improve the performance of algorithms by improving the data quality. Liu [21] vectorized restaurant names and user comments in social networks and improved the low-quality data and data without location labels based on cosine similarity. The performance of the labeling model was improved using game theory [22]. Ju [23] proposed an algorithm for reducing the label noise of labeled medical images based on the Monte Carlo estimation method [24] and a CNN model.

Studies have also been conducted on data valuation algorithms to improve the quality of the training data. Data valuation algorithms evaluate the value of each training sample to be used as its training weight. Leave-one-out (LOO) [25] is the most commonly used data valuation method—it evaluates each sample by adopting the difference between the performance of the model, including and excluding the sample as the sample value. As the computational complexity of LOO increases linearly concerning the number of training samples, it is not suitable for large datasets [25]. Data Shapley [26] is another data valuation algorithm inspired by game theory [22]. It uses marginal performance improvement as the data value after calculating the performance on all possible subsets of the training data. Its computational complexity increases exponentially with the number of training data as it requires training on all possible subsets. Monte Carlo sampling [24] can be used for approximation to reduce the computational complexity. However, it exhibits high computational complexity itself, and approximation introduces certain limitations.

Recently, a meta-learning-based algorithm was proposed that addresses the aforementioned limitations of high computational complexity and approximation. Ren et al. [27] proposed a robust algorithm for low-quality data by adjusting the weight of the batch-size training data for each gradient step using the validation set. Hendrycks [28] corrected the labels of corrupted label data using a clean validation set and re-trained the model using the corrected training data. Saeed [29] used a neural network-based task predictor for image segmentation and classification to update the neural network-based image quality assessment (IQA) controller for medical image data. The authors performed meta-reinforcement learning for newly added data or meta-task data to fine-tune the IQA controller network using the task performance of the predictor. Yoon [30] proposed a deep learning-based data valuation algorithm using reinforcement learning by combining a predictor with a data value estimator (DVE). The DVE was trained using meta-reinforcement learning using the task performance of the predictor. This method exhibited better performance and efficiency than LOO [25] and Data Shapley [26]. Previous data valuation algorithm studies [26,29,30] primarily dealt with public image datasets and insufficiently dealt with other types of data, such as time series data and data with a small number of samples.

In this paper, we propose a data-valuation algorithm based on meta-reinforcement learning for inertial measurement unit-based human activity recognition (hereinafter, IMU-based HAR) data. The IMU comprises a three-axis acceleration sensor and a three-axis gyroscope sensor and measures the inertia applied to the unit based on the captured motion information. The IMU-based HAR algorithm, which is a type of pattern recognition algorithm, recognizes the type and quality of motion based on the IMU data collected using wearable devices. Previous studies on data valuation algorithms have primarily utilized vision-based public datasets [26,29,30]. However, IMU-based HAR data have not been investigated yet. Unlike [30], which required feature-level input, the proposed algorithm adds a feature extraction structure to the data valuation algorithm, enabling the utilization of raw-level inputs. In the algorithm proposed in [30], a pre-trained model was required to accept feature-level input data, which required training data of sufficient volume and quality. Thus, constructing good pre-trained models may be difficult in some cases. The proposed algorithm does not suffer from this limitation. Meanwhile, in previous studies, pre-trained models were used to generate feature-level data to train the network for prediction. However, if a feature extraction structure is included in the data valuation algorithm, as in the proposed algorithm, its network is trained for the purpose of data valuation.

The proposed data valuation algorithm incorporates a structure suitable for IMU-based HAR data within it. It comprises a DVE that receives the training data input and a predictor that supervises the output of the DVE during model training. A feature extraction structure suitable for IMU-based HAR data is added to the DVE to enable it to accept raw-level inputs. Both local and global features can be extracted from the IMU-based HAR data using the feature extraction structure, where a transformer encoder is inserted after the 1D-CNN backbone. A stacking ensemble structure, including a double-head 1D-CNN, which exhibits good performance and efficiency on IMU-based HAR data, is used as the predictor. Four IMU-based HAR datasets are used for model evaluation—the Berg balance scale (BBS) HAR data collected at Inha University Hospital and the public IMU-based HAR datasets from the University of California, Irvine, human activity recognition using a smartphone dataset (UCI-HAR), wireless sensor data mining (WISDM), and the Physical Activity Monitoring dataset (PAMAP2). Corrupted data are generated by contaminating the labels of 20% of the training data to evaluate the algorithm. On all IMU-based HAR datasets, excellent performance is observed concerning the ratio of finding corrupted data in low-value data, exceeding 96%. In addition, a classification performance is observed to improve on all IMU-based HAR datasets when low-value data are removed from the training data. This indicates that the proposed algorithm evaluates the data adequately.

## 2. Materials and Methods

### 2.1. Proposed Algorithm

#### 2.1.1. Structure of the Proposed Data Valuation Algorithm

**Figure 1:**
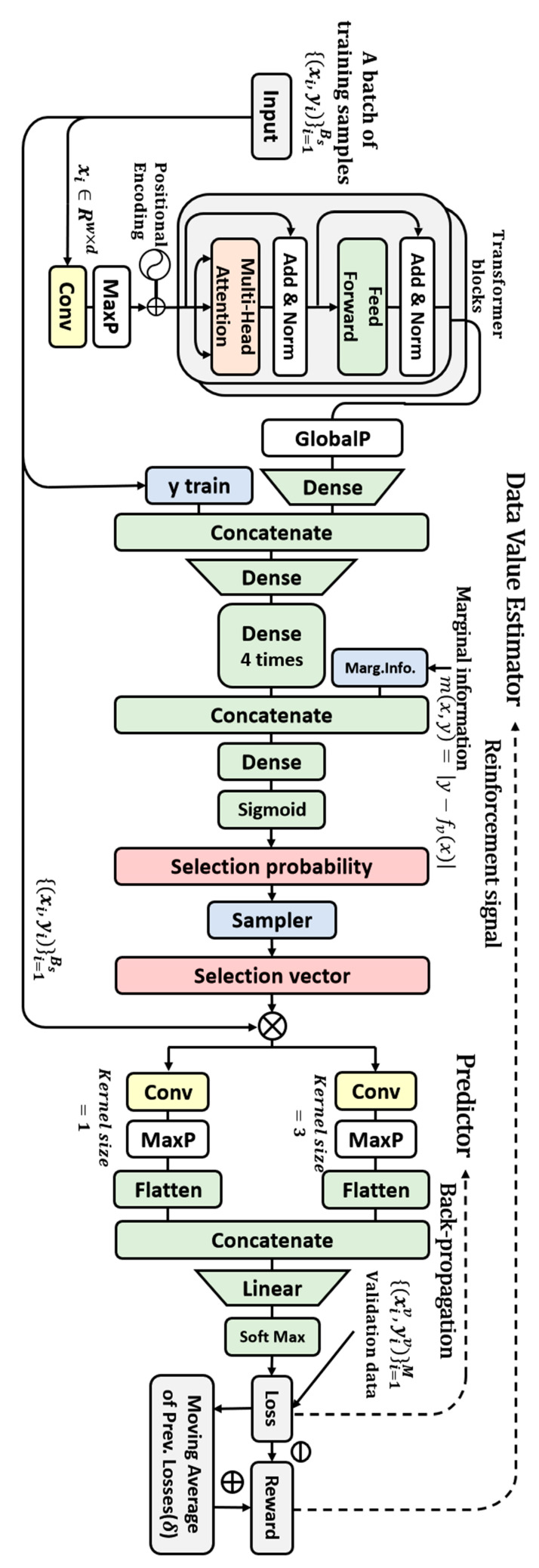
Structure of the proposed algorithm.

The algorithm comprises a DVE and a predictor that supervises the output of the DVE during training. The structure of the algorithm is illustrated in Figure 1.

In the case of a training sample input of batch size = B, the DVE extracts appropriate features from it, concatenates it with label information, and refines the information of the input data using a multi-layer perceptron (MLP) structure comprising five dense layers. All dense layers are structurally identical, with 100 perceptrons and the ReLU activation function. The final dense layer in the MLP is concatenated with marginal information which is the degree of contamination of training data. The marginal information is given by $m\left(x,y\right)=\left|y-f_{v}\left(x\right)\right|$, where $f_{v}$ denotes a predictor pretrained using validation data. Subsequently, a dense layer is placed, and the selection probability, $h_{\varphi }\left(x,y\right)$, of the corresponding data is output as softmax. The selection probability is equal to the value of the corresponding sample. Corresponding to training data, $D={\left\{\left(x,y\right)\right\}}_{i=1}^{N}∼P$, the sampler uses the polynomial distribution $h_{\varphi }\left(D\right)$ obtained by the DVE to choose the selection vector $s=\left\{s_{1},s_{2},\ldots s_{B}\right\}$. The probability of outputting the selection vector, s, is $\pi_{\varphi }\left(D,s\right)=\overset{N}{\underset{i=1}{\prod }}\left[h_{\varphi }{\left(x_{i},y_{i}\right)}^{s}\cdot {\left(1-h_{\varphi }\left(x_{i},y_{i}\right)\right)}^{1-s_{i}}\right]$. The DVE output is passed as a training weight for each sample of the predictor model.

The predictor, $f_{\theta }$, is trained to minimize a weighted loss function, $L_{f}$, on the training dataset, D. Equation (1) express this. Cross-entropy is used as the loss function, and θ denotes a parameter of the predictor model.

(1) $$f_{\theta }=argmin_{f\in F}\;\;\frac{1}{N}\overset{N}{\underset{i=1}{\sum }}h_{\varphi }\left(x_{i},y_{i}\right)\cdot \nabla L_{f}\left({\hat{f}}_{\theta }\left(x_{i}\right),y_{i}\right)$$

The task performance of trained predictor is used as the loss, $L_{h}$. At this time, to calculate the task performance, clean validation data $D^{v}={\left\{\left(x^{v},y^{v}\right)\right\}}_{k=1}^{L}∼P^{t}$ are used. The DVE loss is trained using a gradient-based method. The loss is transmitted to the DVE as a reward. The loss for DVE training obtained by multiplying the probability and reward corresponding to the sample can be expressed as the following expected value:

(2) $$\begin{array}{cc} \hat{l}\left(\varphi \right) & =E_{\left(x^{v},y^{v}\right)∼P^{t},s∼\pi_{\varphi }\left(D,\cdot \right)}\left[L_{h}\left(f_{\theta }\left(x^{v}\right),y^{v}\right)\right]\\ & =\int P^{t}\left(x^{v}\right)\underset{s\epsilon {\left[0,1\right]}^{N}}{\sum }\pi_{\varphi }\left(D,s\right)\cdot \left[L_{h}\left(f_{\theta }\left(x^{v}\right),y^{v}\right)\right]dx^{v} \end{array}$$

At this time, the agent is DVE, the action is the data selection process, and the environment encompasses training and evaluation of the predictor.

#### 2.1.2. Structure for IMU-Based HAR in Proposed Data Valuation Algorithm

The proposed data valuation algorithm introduces appropriate deep learning structures into an existing algorithm [30] to achieve a good performance on IMU-based HAR data. A structure for extracting the features of IMU-based HAR data is inserted into the data input part of the DVE to enable raw-level, IMU-based HAR data to be accepted as input. The predictor uses a module with good efficiency and performance on IMU-based HAR data.

The feature extraction structure extracts both local and global features by inserting a transformer encoder after the 1D-CNN backbone. The filter size of the 1D-CNN layer is taken to be 64, its kernel size is taken to be 3, and the GeLU activation function is used. Two self-attention heads are used in the transformer block, 256 perceptrons are used in the feed-forward layer, a dropout of 0.1 is used, GeLU is used as the activation function, and two structurally identical transformers are placed in a row. In previous studies on IMU-based HAR data [31,32,33], models comprising a recurrent neural network (RNN) series after the 1D-CNN exhibited good performance. The feature extraction structure used in this study is inspired by those used in previous studies [31,32,33]. Rather than using an RNN-series model, a transformer encoder block is used to perform a similar role. The latter is superior to the former in terms of computational efficiency [34].

A 1D-CNN-based stacking ensemble structure model [35,36,37,38] that exhibits good performance and efficiency in an inertial sensor-based HAR algorithm is used as the predictor. Structurally, it consists of a simple dense layer classifier after a double-head 1D-CNN, and the kernel sizes of the two heads are taken to be 1 and 3 to extract different features. The filter size of the 1D CNN layer is taken to be 64, and ReLU is used as the activation function.

### 2.2. Evaluation Datasets

In this study, four IMU-based HAR datasets are used to evaluate the proposed algorithm—the BBS HAR data collected together with the Department of Rehabilitation Medicine at Inha University Hospital and public datasets, namely, UCI-HAR, WISDM, and PAMAP2 data.

#### 2.2.1. BBS HAR Data

The BBS HAR dataset comprises IMU-based HAR data recorded by introducing a wearable inertial measurement unit (IMU) into a BBS, a balanced assessment. The BBS is the balance assessment designed to evaluate the balance ability of the elderly and is known to be highly reliable, even for patients with brain diseases [39,40]. In BBS, subjects are asked to perform 14 static and dynamic tasks, each of which is scored. The balance ability of the subject is evaluated on the basis of the total score [41].

The data were collected from the Department of Rehabilitation Medicine at Inha University Hospital. The experimental design was approved by the Institutional Review Board. In aggregate, 53 patients aged 50–80 years (male: 31, female: 22) with brain disease and three healthy individuals in their late 20s participated in the experiment. The healthy participants imitated the motions of the patients and performed all motions with scores between 0 and 4.

**Figure 2:**
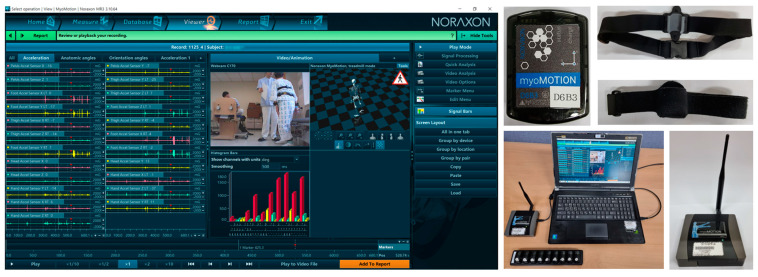
Software and equipment of the multichannel wireless IMU system.

Noraxon’s myoMotion, which is a multichannel wireless IMU system, is used for the experiment. This system is certified to be an ISO 13,485 compliant (Registration # MED−0037b) and an FDA 510 K compliant (Registration number #2098416) medical device. IMU modules are attached to the human body using Velcro bands. The IMU modules transmit data wirelessly to receivers, which are connected to a computer via a USB. The system uses a type of PC software for recording and management hardware. If a webcam is connected to the PC, video data synchronized with IMU data can be recorded, which can be used to label IMU motion data. Figure 2 illustrates the software and equipment of the Noraxon’s myoMotion.

**Figure 3:**
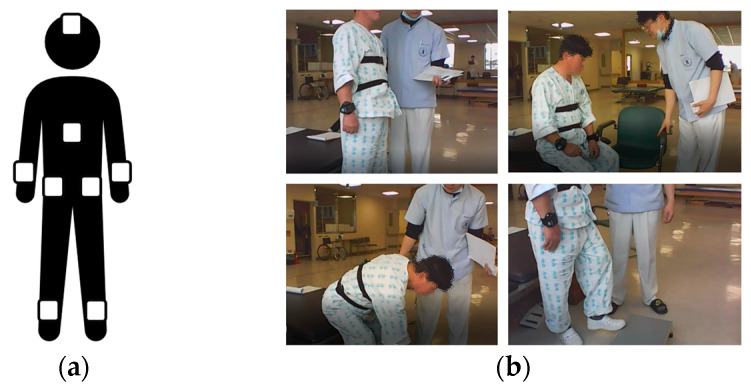
IMU attachment locations and photograph of experiment: (**a**) IMU attachment locations; (**b**) photograph of the BBS experiment.

Eight IMUs are used in the experiment and worn on the forehead, left and right wrists, left and right upper hips, back of the left and right ankles, and back. Each IMU recorded the values of accelerometer X, Y, Z, roll, pitch, yaw, rotation X, Y, Z at a sampling rate of 100 Hz. The rotation refers to the number of rotations and is calculated by cumulatively aggregating the rotation angles of the sensor. Figure 3 depicts a photograph of the BBS experimental environment and the IMU attachment locations.

In [35], a deep-learning-based BBS score recognition algorithm was proposed. We adopt the data pre-processing methodology of the aforementioned algorithm, comprising data augmentation based on the oversampling technique, data downsampling, normalization, and zero-padding [35].

#### 2.2.2. UCI-HAR

The UCI-HAR dataset comprises IMU-based HAR data obtained using inertial sensors embedded in smartphones and were devised by Anguita [42]. The participants performed six motions in aggregate—“walking”, “walking upstairs”, “walking downstairs”, “sitting”, “standing”, and “lying down”—while wearing a smartphone on their waist. A total of 30 participants aged 19–48 years participated in the experiment. Motion data were recorded using a 3-axis gyroscope and 3-axis accelerometer at a sampling rate of 50 Hz. A sliding window was applied to the data for real-time recognition. The window size was taken to be 128, with an overlap of 50%. Data augmentation was performed using the same method as that in a previous study [37].

#### 2.2.3. WISDM

The WISDM dataset comprises HAR data obtained using inertial sensors embedded in smartphones and were devised by Kwapisz [43]. Each participant performed six movements—“walking”, “jogging”, “ascending stairs”, “descending stairs”, “sitting”, and “standing”—with a smartphone in the front pocket of their trousers. A total of 36 people participated in the experiment, and the 3-axis accelerometer data were recorded at a sampling rate of 20 Hz. For real-time recognition, a sliding window was applied to WISDM data with a window size of 80 and an overlap of 50%. Data augmentation was performed using the same method as in a previous study [37].

#### 2.2.4. PAMAP2

The PAMAP2 dataset comprises IMU-based HAR data collected by Reiss [44] from test participants using three wearable IMUs on their hands, chest, and ankles and a heart rate sensor. The test participants performed 12 movements commonly performed in daily life—“lying”, “sitting”, “standing”, “walking”, “running”, “cycling”, “Nordic walking”, “ascending stairs”, “ descending stairs”, “vacuum cleaning”, “ironing”, and “rope jumping”—and six optional movements—“watching TV”, “computer work”, “car driving”, “folding laundry”, “house cleaning,” and “playing soccer.” In this study, 12 types of data corresponding to actions undertaken in daily life are used. Nine participants aged 27–32 years participated in the test. The 3-axis gyroscope, 3-axis accelerometer, and 3-axis geomagnetic and temperature sensor data were recorded at a sampling rate of 100 Hz, and the heart rate data were recorded at a sampling rate of 9 Hz. For real-time recognition, a sliding window was applied to the data with a window size of 100 and an overlap of 50%. Data augmentation was performed in the same manner as in a previous study [45], and synthetic data were generated to ensure at least 6500 windowed data for each class.

### 2.3. Training and Evaluation Method

For the evaluation of the proposed algorithm, the data are divided into training, validation, and test datasets in a 4:2:3 ratio. The predictor pre-trains with training data. At this time, the batch-size training data is received from the DVE. The batch size of the predictor is considered as 64 for BBS data and 1024 for public data based on excellent results obtained and corresponding to the batch sizes [35,37]. The predictor uses the Adam optimizer, a learning rate of 0.01, and 200 iterations. DVE uses the reward received from the predictor for training. The DVE uses the Adam optimizer, a learning rate of 0.01, and 30 iterations for training. Algorithm 1 describes the training process of the data valuation algorithm using a pseudocode.

**Algorithm 1** Pseudo-code of data valuation training

$\mathrm{Inputs}:\;\mathrm{Learning}\;\mathrm{rates}\;\alpha ,\beta >0,\;\mathrm{mini-batch}\;\mathrm{size}\;B_{p},B_{s}>0,\;\mathrm{inner}\;\mathrm{iteration}\;\mathrm{count}\;N_{I}>0,\;\mathrm{moving}\;\mathrm{average}\;\mathrm{window}\;T>0,\;\mathrm{training}\;\mathrm{dataset}\;D,\;\mathrm{validation}\;\mathrm{dataset}\;D^{v}={\left\{\left(x_{k}^{v},y_{k}^{v}\right)\right\}}_{k=1}^{L}$



Initialize parameters θ, φ, moving average δ=0



While waiting for convergence perform



$\;\mathrm{Sample}\;D_{B}={\left(x_{j},y_{j}\right)}_{j=1}^{B_{s}}∼D$



$\;\mathrm{for}\;j=1,\;\ldots ,\;B_{s}\;\mathrm{perform}$



$\;\;\mathrm{Get}\;\mathrm{selection}\;\mathrm{probabilities}:\;w_{j}=h_{\varphi }\left(x_{j},\;y_{j}\right)$



$\;\;\mathrm{Sample}\;a\;\mathrm{selection}\;\mathrm{vector}:\;s_{j}∼Ber\left(w_{j}\right)$



$\;\mathrm{for}\;t=1,\;\ldots ,\;N_{I}\;\mathrm{perform}$



$\;\;\mathrm{Sample}\;{\left({\tilde{x}}_{m},{\tilde{y}}_{m},{\tilde{s}}_{m}\right)}_{m=1}^{B_{p}}∼{\left(x_{j},y_{m},s_{m}\right)}_{j=1}^{B_{s}}$



  Update the predictor model:



$\;\;\;\theta \leftarrow \theta -\frac{\alpha }{B_{p}}\overset{B_{p}}{\underset{m=1}{\sum }}{\tilde{s}}_{m}\cdot \nabla_{\theta }L_{f}\left(f_{\theta }\left({\tilde{x}}_{m}\right),{\tilde{y}}_{m}\right)$

 Update the DVE model:

$\;\;\varphi \leftarrow \varphi -\left[\frac{B}{L}\overset{L}{\underset{k=1}{\sum }}\left[L_{h}\left(f_{\theta }\left(x_{k}^{v}\right),y_{k}^{v}\right)\right]-\delta \right]\cdot \nabla_{\varphi }log\pi_{\varphi }\left(D_{B},\left(s_{1},\ldots ,s_{b}\right)\right)$

 Update the baseline:

$\;\;\;\;\;\;\delta \leftarrow \frac{T-1}{T}\delta +\frac{1}{LT}\overset{L}{\underset{k=1}{\sum }}\left[L_{h}\left(f_{\varphi }\left(x_{k}^{v}\right),y_{k}^{v}\right)\right]$



Corrupted sample discovery (CSD) and remove high/low-value samples (RHLVS) are used to evaluate the data valuation algorithm. In total, 20% of the labels of the training data are contaminated for evaluation. CSD represents the rate at which corrupted samples are discovered while accumulating a constant rate of the amount of data from the lowest value data. The performance of the data valuation algorithm can be considered to be excellent when several corrupted samples are observed in the low-value data. In the previous studies on data valuation algorithms [26,30], CSD was used as a performance evaluation criterion. A value is assigned to each sample of the training dataset using the trained data valuation algorithm, and the dataset is in descending order in terms of the value of $D^{sortH}={\left\{\left(x_{i}^{h},y_{i}^{h}\right)\right\}}_{i=1}^{N}$. The contaminated training dataset is denoted by $D^{conta}={\left\{\left(x,y\right)\right\}}_{i=1}^{o}$. The number of data corresponding to 5% of the training data is $v=\frac{N}{100/5}$, and the index of the result is r ϵ {0,1,… 10}. Accumulated data with low values are denoted by $D_{r}^{sortH}={\left\{\left(x_{i}^{H},y_{i}^{H}\right)\right\}}_{i=1}^{v\times r}$. The function that outputs the number of elements in a dataset is denoted by Numb. The formula for obtaining CSD and the resulting value can be expressed as follows:

(3) $$\begin{array}{c} {\mathrm{CSD}}_{r}=\frac{Numb(D_{r}^{sortH}\cap {\;D}^{conta})}{o}\\ \mathrm{CSD}=\left\{CSD_{0},\ldots ,\;CSD_{10}\right\} \end{array}$$

RHLVS repeatedly removes a certain amount of data—either of highest or lowest value—and evaluates the accuracies of HAR after removing each training data point. If the data valuation model exhibits excellent performance, the classification accuracy can be decreased by removing high-value data, and the classification accuracy can be slightly improved by removing low-value data. The value (=selection probability) of each training sample calculated using the data valuation algorithm is given by $h_{\varphi }\left(D\right)=\left\{p_{1},\ldots ,p_{N}\right\}$. When $Sort_{h}$ is a descending sort function and $Sort_{L}$ is an ascending sort function, $Sort_{h}\left(h_{\varphi }\left(D\right)\right)=\left\{p_{1}^{h}{}_{1},\ldots ,p_{N}^{h}\right\}$, $Sort_{L}\left(h_{\varphi }\left(D\right)\right)=\left\{p_{1}^{l},\ldots ,p_{N}^{l}\right\}$. The sorted training data are denoted by $D^{sortL}={\left\{\left(x_{i}^{l},y_{i}^{l}\right)\right\}}_{i=1}^{N}$ and $D^{sortH}={\left\{\left(x_{i}^{h},y_{i}^{h}\right)\right\}}_{i=1}^{N}$. The classification model is denoted by $C_{\gamma }$, where γ denotes the training parameter. The accuracy function, Accuracy, calculates the accuracy using prediction labels and true test labels. The formulas and results used to obtain “remove high value data” and “remove low value data” can be expressed as follows:

(4) $$\begin{array}{c} C_{\gamma r}^{h}=argmin_{f\in F}\frac{1}{N-v*r}\overset{N}{\underset{i=v\times r}{\sum }}p_{i}^{h}\cdot \nabla L_{C}\left({\hat{C}}_{\gamma }\left(x_{i}^{h}\right),y_{i}^{h}\right)\\ C_{\gamma r}^{l}=argmin_{f\in F}\frac{1}{N-v*r}\overset{N}{\underset{i=v\times r}{\sum }}p_{i}^{l}\cdot \nabla L_{C}\left({\hat{C}}_{\gamma }\left(x_{i}^{l}\right),y_{i}^{l}\right)\\ Acc_{r}^{h}=Accuracy\left(C_{\gamma r}^{h}\left(D^{t}\right),y^{t}\right)\\ Acc_{r}^{l}=Accuracy\left(C_{\gamma r}^{l}\left(D^{t}\right),y^{t}\right)\\ remove\;high\;value\;data=\left[Acc_{0}^{h},\ldots ,Acc_{10}^{h}\right]\\ remove\;low\;value\;data=\left[Acc_{0}^{l},\ldots ,Acc_{10}^{l}\right] \end{array}$$

**Figure 4:**
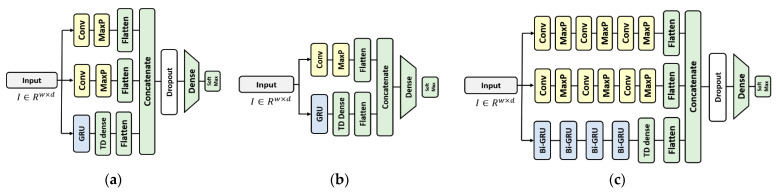
Deep learning-based HAR model for performance evaluation of the data valuation algorithm: (**a**) the model for BBS HAR data, (**b**) baseline model for public HAR data, and (**c**) complex model for public HAR data.

For RHLVS, a deep learning-based classification model is used. A model structurally identical to the model for BBS HAR data proposed in [35] but with a slightly lower capacity is used for the BBS HAR data. The complex model for public HAR data proposed in [36] is used for public data, and the baseline model for public HAR data is used for comparison. Figure 4 depicts the structure of the HAR model for BBS and public data. The 1D-CNN convolutional layer used in the three models has a filter size = 64, kernel size = 3, activation function = “ReLU”, and maxpooling size = 2. The unit size of the GRU layer is taken to be eight, and an 8-size hidden state is the output for each unit. The Model for BBS HAR data uses a 50% dropout layer, and the complex model for public HAR data uses a 70% dropout layer. The number of perceptrons in the dense layer is 100 in all three models.

## 3. Results and Discussion

### 3.1. Evaluation of the Proposed Algorithm on BBS HAR Data

**Figure 5:**
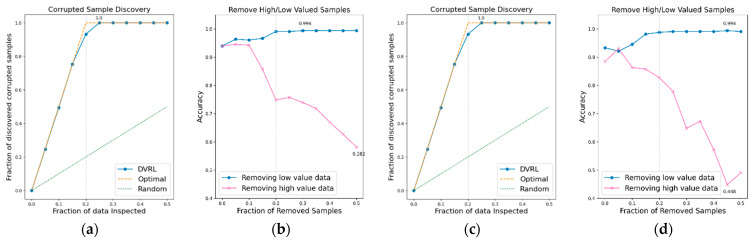
CSD and RHLVS on BBS HAR data: (**a**) CSD graph of BBS task 1, (**b**) RHLVS graph of BBS task 1, (**c**) CSD graph of BBS task 2, and (**d**) RHLVS graph of BBS task 2.

**Figure 6:**
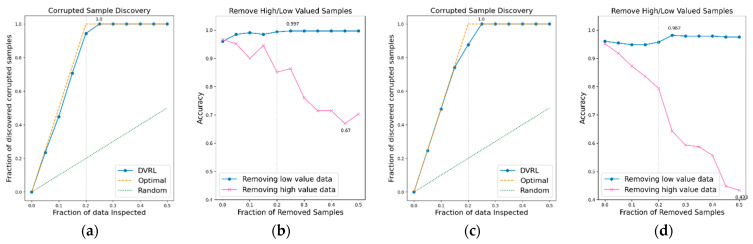
CSD and RHLVS on BBS HAR data: (**a**) CSD graph of BBS task 3, (**b**) RHLVS graph of BBS task 3, (**c**) CSD graph of BBS task 4, and (**d**) RHLVS graph of BBS task 4.

**Figure 7:**
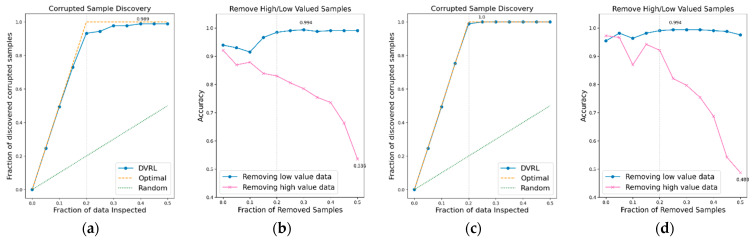
CSD and RHLVS on BBS HAR data: (**a**) CSD graph of BBS task 5, (**b**) RHLVS graph of BBS task 5, (**c**) CSD graph of BBS task 6, and (**d**) RHLVS graph of BBS task 6.

**Figure 8:**
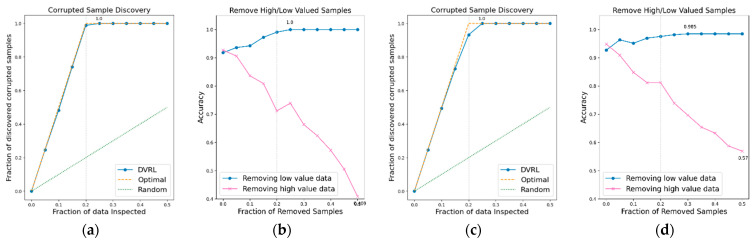
CSD and RHLVS on BBS HAR data: (**a**) CSD graph of BBS task 7, (**b**) RHLVS graph of BBS task 7, (**c**) CSD graph of BBS task 8, and (**d**) RHLVS graph of BBS task 8.

**Figure 9:**
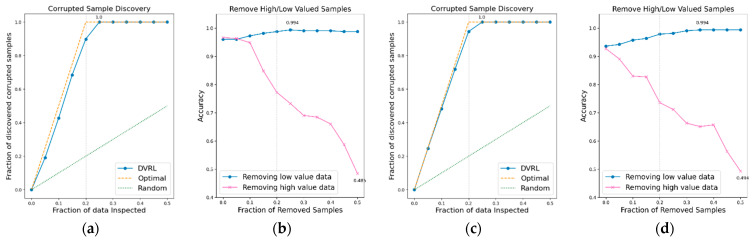
CSD and RHLVS on BBS HAR data: (**a**) CSD graph of BBS task 9, (**b**) RHLVS graph of BBS task 9, (**c**) CSD graph of BBS task 10, and (**d**) RHLVS graph of BBS task 10.

**Figure 10:**
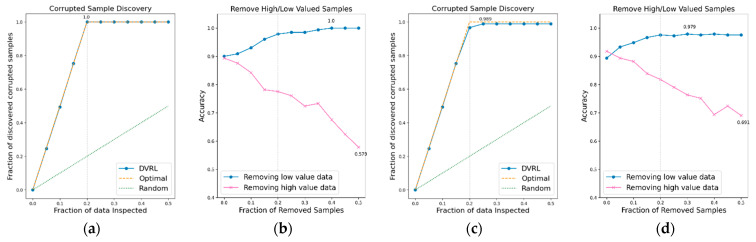
CSD and RHLVS on BBS HAR data: (**a**) CSD graph of BBS task 11, (**b**) RHLVS graph of BBS task 11, (**c**) CSD graph of BBS task 12, and (**d**) RHLVS graph of BBS task 12.

**Figure 11:**
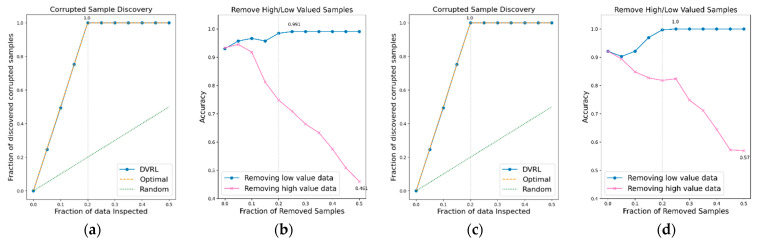
CSD and RHLVS on BBS HAR data: (**a**) CSD graph of BBS task 13, (**b**) RHLVS graph of BBS task 13, (**c**) CSD graph of BBS task 14, and (**d**) RHLVS graph of BBS task 14.

The performance of the data valuation algorithm is evaluated through CSD and RHLVS after training the algorithm using the BBS HAR data. Figure 5, Figure 6, Figure 7, Figure 8, Figure 9, Figure 10 and Figure 11 depict the CSD and RHLVS of the proposed algorithm on the BBS HAR data. In CSD, the maximum corrupted discovery rate is plotted on a graph. In RHLVS, the maximum and minimum accuracies are indicated on the graph.

In the CSD graphs depicted in Figure 5a,c, Figure 6a,c, Figure 7a,c, Figure 8a,c, Figure 9a,c, Figure 10a,c and Figure 11a,c, the optimal graph corresponding to ideal model performance is drawn. Greater amounts of contaminated data in the low-value data correspond to higher proximity between the algorithm’s performance graph and the optimal graph. All data graphs depicted in BBS tasks 1–14 are confirmed to be close to the optimal graph, corroborating the excellent performance of the algorithm. The RHLVS graphs depicted in Figure 5b,d, Figure 6b,d, Figure 7b,d, Figure 8b,d, Figure 9b,d, Figure 10b,d and Figure 11b,d indicate that, in the remove high value data graph, the accuracy decreases significantly as the percentage of data to be removed increases. Meanwhile, in the remove low value data graph, the reduction in accuracy is small even when the percentage of data to be removed is high. As this tendency is clear, the performance of the data valuation algorithm is considered to be good. The performance is improved by removing low-value data from all BBS data. Thus, the data valuation algorithm improves classification performance by improving the training data quality.

Table 1 summarizes the major results obtained for RHLVS and CSD on BBS data, including maximum accuracy, improved accuracy, and removed data. Improved accuracy indicates the maximum improved performance achieved by removing low-value data. Removed data represents the rate of removal of low-value data at maximum accuracy. For CSD, the major metrics are maximum discovery and removed data. Maximum discovery represents the ratio of the corrupted data when the discovery of corrupted data is the maximum. Removed data represents the ratio of removed low-value data when the discovery of corrupted data is the maximum.

The CSD values on BBS tasks 1–14 indicate that the average maximum discovery is 99.8%, and the average removed data is 25%. As the corrupted data comprises 20% of the training data, the CSD performance is almost ideal. The RHLVS values indicate that the average maximum accuracy is 99.3%, which corresponds to excellent performance, and the average improved accuracy is 5.9%. Futhermore, a performance improvement is confirmed in all the tasks. Data with values below 25% are observed to be primarily composed of contaminated data. Therefore, the performance improvement observed in the initial part of the remove low value data graph seems to be primarily caused by the removal of contaminated data. As the average removed data for RHLVS is 29.5% and the that for CSD is 25%, data with a low value among the clean data are also removed in the former case.

### 3.2. Evaluation of the Proposed Algorithm on BBS HAR Data

The primary purpose of the data valuation algorithm is to improve classification performance by enhancing the quality of training data. In this study, performance improvement is confirmed by removing low-value data from the training data in RHLVS using the proposed data valuation algorithm. A comparison of the results of this study with those of a previous study on BBS HAR [35] reveals the extent of improvement. This study uses the same data and a structurally identical model with a slightly smaller capacity as [35]. Table 2 depicts the performance reported in [35], the maximum accuracy calculated from the RHLVS in this study, and the volume of training data used for training. In this study, 44% of the total data are used as training data, which is determined by accounting for the ratio of the removed data at the maximum accuracy for RHLVS.

By improving the corrupted BBS HAR data by applying the data valuation algorithm, the proposed algorithm outperformed the method proposed in [35], which used clean data. The application of the proposed data valuation algorithm improves performance perceptibly as corrupted training data as well as low-value data are removed from the clean data. Moreover, excellent performance is confirmed when a small quantity of high-quality data is used—the algorithm proposed in this study uses approximately 59% less training data on average than that of [35].

### 3.3. Evaluation of the Proposed Algorithm on Public HAR Data

**Figure 12:**
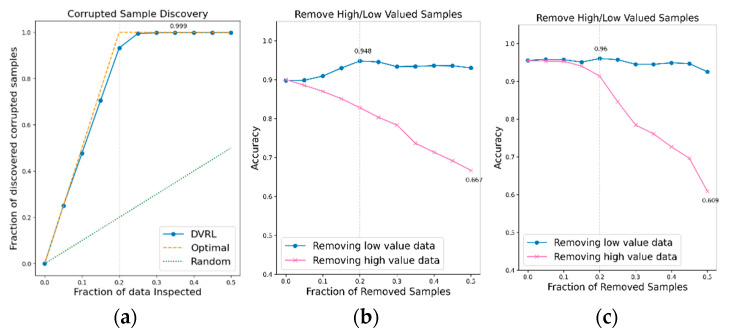
CSD and RHLVS on IMU-based HAR public data: (**a**) CSD graph of UCI-HAR data, (**b**) RHLVS graph of UCI-HAR data using baseline model for public HAR data, and (**c**) RHLVS graph of UCI-HAR data using the complex model for public HAR data.

**Figure 13:**
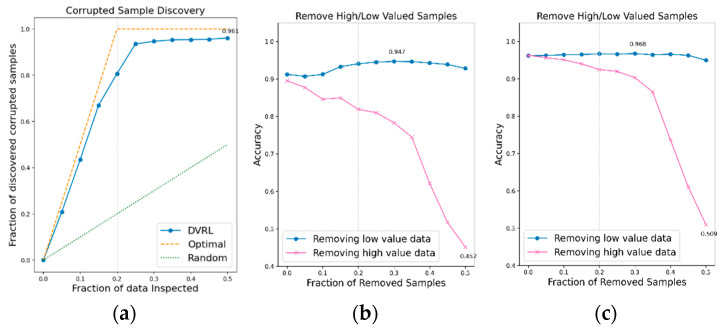
CSD and RHLVS on IMU-based HAR public data: (**a**) CSD graph of WISDM data, (**b**) RHLVS graph of WISDM data using baseline model for public HAR data, and (**c**) RHLVS graph of WISDM data using the complex model for public HAR data.

**Figure 14:**
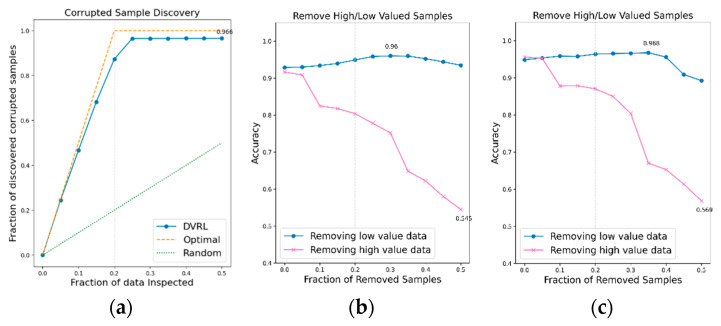
CSD and RHLVS on IMU-based HAR public data: (**a**) CSD graph of PAMAP2 data, (**b**) RHLVS graph of PAMAP2 data using baseline model for public HAR data, and (**c**) RHLVS graph of PAMAP2 data using the complex model for public HAR data.

An additional experiment is conducted to verify if the proposed data valuation algorithm, which exhibits good performance on BBS HAR data, continues to perform well on public IMU-based HAR data. The data valuation algorithm is applied to public IMU-based HAR datasets, UCI-HAR, WISDM, and PAMAP2, and its performance is evaluated in terms of CSD and RHLVS. The baseline model for public HAR data and complex model for public HAR data are used to evaluate the performance of the data valuation algorithm on public IMU-based HAR data. Figure 12, Figure 13 and Figure 14 depict the CSD and RHLVS results on public HAR data. In the case of CSD, the maximum corrupted discovery rate is plotted on the graph. In the case of RHLVS, the maximum and minimum accuracies are indicated on the graph and the baseline, and complex model for public HAR data are used for classification.

Figure 12a,c, Figure 13a,c and Figure 14a,c indicate that the CSD graphs are close to the optimal graphs on all public HAR datasets, and the performance of the algorithm is good in all cases, despite being inferior to that of BBS data. Figure 12b,d, Figure 13b,d and Figure 14b,d indicate that the accuracy of the removed high-value data graph decreases significantly as the percentage of data removed increases. On the other hand, in the removed low-value data graph, the reduction in accuracy is small even when the percentage of data removed is high. As this tendency is clear, the data valuation algorithm can be considered to perform well. In the removed low- value data graph, the performance improvement is attributed to the removal of low-value data. Thus, the performance of the classification model is improved by improving the quality of training data using the proposed data valuation algorithm.

Table 3 presents the major metrics of RHLVS and CSD. For RHLVS, these are the maximum accuracy, improved accuracy, and removed data. Improved accuracy represents the maximum improved performance while removing low-value data. The removed data represents the rate of removal of low-value data at maximum accuracy. The major metrics of CSD are maximum discovery and removed data. The maximum discovery represents the ratio of corrupted data when the discovery of corrupted data is at its maximum. The removed data represents the ratio of the removed low-value data when the discovery of corrupted data is at its maximum.

The CSD on the IMU-based HAR data reveals that more than 96% of the contaminated data is identified on all IMU-based HAR datasets. Maximum discovery and the removed data of CSD are observed to be 35%, 50%, and 50% of the UCI-HAR, WISDM, and PAMAP2 data, respectively. However, when 25% of the low-value data are accumulated in all three datasets, the discovery rate of corrupted data becomes close to the maximum discovery rate. Considering that corrupted data accounts for 20% of the training data, the CSD performance can be considered to be excellent. In RHLVS, the maximum accuracies in the baseline model for public HAR data are 94.8%, 94.7%, and 96.0% for the UCI-HAR, WISDM, and PAMAP2 datasets, respectively, and 96.0%, 96.8%, and 96.8% for the complex model for public HAR data. The accuracy is improved by 5%, 3.5%, and 3.1% on the UCI-HAR, WISDM, and PAMAP2 datasets, respectively, when using the Baseline model for public HAR data, and by 0.4%, 0.6%, and 1.9%, respectively, when using the Complex model for public HAR data. Performance improvement is confirmed in all experiments by improving the data quality using the data valuation algorithm. The improvement in accuracy over the baseline model for public HAR data is greater than that over the complex model for public HAR data owing to the better regularization performance of the latter. In RHLVS, the removed data value is 20%, 30%, and 50% for UCI-HAR, WISDM, and PAMAP2 data, respectively. When 25% of the low-value data is removed, the CSD performance is observed to be almost saturated on all three datasets. As the number of training data gradually decreases as the data are removed, it seems that the maximum accuracy is attained before reaching the maximum discovery. In conclusion, the proposed data-valuation algorithm is observed to exhibit excellent classification performance on IMU-based HAR public data.

## 4. Conclusions

In this paper, a meta-reinforcement learning-based data-valuation algorithm was proposed to improve the IMU-based HAR training data. A deep learning structure suitable for IMU-based HAR was introduced in the DVE, and a predictor was added to construct the data valuation algorithm. In previous studies [26,29,30], vision-related public datasets were used primarily, because the purpose of the data valuation algorithm was limited to improve image classification. In this study, HAR data, which is multivariate timeseries data derived from human movements, is targeted. In general, HAR data has a lower resolution than vision data, and the amount of data in a public dataset is relatively very small. The purpose of our study is to find a good data valuation algorithm that functions efficiently on HAR data. The proposed data valuation algorithm improves the DVE structure used in previous studies [30], which were not capable of feature extraction after adding this capability. Therefore, the proposed algorithm can accept raw-level data as an input, making pre-training redundant. In previous studies, a pre-trained model was used to train the network for prediction purposes. However, the feature extraction network of the proposed algorithm was trained explicitly for data valuation. As the feature extraction structure, a transformer encoder block was inserted after the 1D-CNN backbone in front of the DVE, enabling the extraction of both local and global features. The proposed data-valuation algorithm is observed to be capable of performing feature extraction and data-value estimation simultaneously. The predictor uses a multi-head 1D-CNN-based stacking ensemble structure with good efficiency and performance on IMU-based HAR data. Two metrics, CSD and RHLVS, were used to evaluate the algorithm. In terms of CSD, the ability of the algorithm to discover corrupted data is observed to be excellent on all four IMU-based HAR datasets. In particular, the ability to identify corrupted BBS HAR data is nearly ideal. In terms of RHLVS, the performance is observed to be improved by removing low-value data. The proposed data valuation algorithm exhibits excellent performance in assigning data values to all IMU-based HAR data, confirming that it can contribute to the improvement of the quality of IMU-based HAR data and HAR model performance.

The proposed data valuation algorithm suffers from the limitation of requiring manual updating of clean validation data when new data are added, which can be time-consuming and effort-intensive. We intend to compensate for this limitation in a follow-up study by utilizing a semi-supervised learning algorithm to remove outliers and identify good-quality validation data. Another limitation of the proposed data valuation algorithm is that its entire structure cannot use gradient descent or backpropagation, which is commonly used for training deep neural network algorithms, as its sampler structure is non-differentiable. This is why the algorithm uses meta-reinforcement learning instead. In a follow-up study, we intend to use an alternative differentiable structure, enabling the algorithm to be trained using gradient descent or backpropagation. This may improve training efficiency and performance.

Recently, large technology companies have started to gather smartphone and smart watch-based healthcare data automatically to provide more comprehensive healthcare service. These data require management and quality control to ensure good service. We expect the proposed algorithm to be efficient and effective in this regard.

## Figures and Tables

**Table 1:** Major metrics of data valuation algorithm on BBS data.

Task	Major Figures of RHLVS (%)			Major Figures of CSD (%)	
	Maximum Accuracy	Improved Accuracy	Removed Data	Maximum Discovery	Removed Data
1	99.4	5.5	30	100	25
2	99.4	6.1	45	100	25
3	99.7	3.6	25	100	25
4	98.2	2.1	25	100	25
5	99.4	5.5	30	98.9	40
6	99.4	3.9	25	100	25
7	100	8.2	25	100	25
8	98.5	5.8	30	100	25
9	99.4	3.3	25	100	25
10	99.4	5.8	35	100	25
11	100	10	40	100	20
12	97.9	8.5	30	98.9	25
13	99.1	6.1	25	100	20
14	100	7.9	25	100	20
Average	99.3	5.9	29.5	99.8	25

**Table 2:** Comparison of BBS scoring performances of [35] and after applying the DVRL algorithm.

Task	Accuracy (%)		Amount of Training Data (%)	
	Previous Study [35]	This Study	Previous Study [35]	This Study
1	98.5	99.4	90	30.8
2	98.5	99.4	90	24.2
3	99.6	99.7	90	33
4	99	98.2	90	33
5	96.7	99.4	90	30.8
6	97.9	99.4	90	33
7	99	100	90	33
8	98.9	98.5	90	30.8
9	97.8	99.4	90	33
10	98.2	99.4	90	28.6
11	97.8	100	90	26.4
12	98.2	97.9	90	30.8
13	98.1	99.1	90	33
14	99.1	100	90	33
Average	98.4	99.3	90	31

**Table 3:** Major metrics used to evaluate the proposed data valuation algorithm on public IMU-based HAR data.

Data	Major Metrics of RHLVS Baseline Model for Public HAR Data (%)			Major Metrics of RHLVS Complex Model for Public HAR Data (%)			Major Metrics of CSD (%)	
	Maximum Accuracy	Improved Accuracy	Removed Data	Maximum Accuracy	Improved Accuracy	Removed Data	Maximum Discovery	Removed Data
UCI-HAR	94.8	5	20	96.0	0.4	20	99.9	35
WISDM	94.7	3.5	30	96.8	0.6	30	96.1	50
PAMAP2	96.0	3.1	30	96.8	1.9	35	96.6	50

## Data Availability

The experiments have been carried out using sensor-based HAR datasets such as UCI [42], WISDM [43] and PAMAP2 [44] which are open for use in the research work.
